# The Effectiveness and Safety of Autologous Platelet Concentrates as Hemostatic Agents after Tooth Extraction in Patients on Anticoagulant Therapy: A Systematic Review of Randomized, Controlled Trials

**DOI:** 10.3390/jcm12165342

**Published:** 2023-08-17

**Authors:** Maria Domenica Campana, Angelo Aliberti, Alfonso Acerra, Pasquale Sammartino, Pasquale Dolce, Gilberto Sammartino, Roberta Gasparro

**Affiliations:** 1 Department of Neurosciences, Reproductive Sciences and Odontostomatological Sciences, University of Naples Federico II, 80131 Naples, Italy; mar.campana@studenti.unina.it (M.D.C.); ange.aliberti@studenti.unina.it (A.A.); alf.acerra@studenti.unina.it (A.A.); roberta.gasparro@unina.it (R.G.); 2 Multidisciplinary Department of Medical-Surgical and Odontostomatological Specialties, University of Campania “Luigi Vanvitelli”, 80138 Naples, Italy; pasqualesammartino91@gmail.com; 3 Department of Public Health, University of Naples Federico II, 80131 Naples, Italy; pasquale.dolce84@gmail.com

**Keywords:** anticoagulant therapy, time to hemostasis, healing, platelet-rich fibrin, autologous platelet concentrates, post-operative bleeding, quality of life, dental extraction, hemostatic agents

## Abstract

One of the common challenges in oral surgery is dealing with patients who are taking oral anticoagulant/antiaggregant drugs. Several local hemostatic agents have been proposed as an alternative to conventional suturing. Among these, autologous platelet concentrates (APCs) have been widely used to decrease the risk of hemorrhage after dental extraction. Nevertheless, there is a lack of consensus regarding the superiority of any one specific hemostatic agent over the others. This systematic review is aimed at evaluating the effectiveness of APCs as hemostatic agents after tooth extraction in patients on anticoagulant therapy. A literature search was conducted of articles published before March 2023 on PubMed, Scopus, and the Cochrane Central Register of Controlled Trials (CENTRAL). Studies on the use of APCs in patients undergoing dental extractions and being treated with anticoagulant drugs were included. Only randomized, controlled trials (RCTs) published up to March 2023 were included; the outcomes assessed were the time to hemostasis, the presence of post-operative bleeding and pain, and the effectiveness of wound healing. The risk of bias for each RCT was assessed by using the ‘risk of bias’ tool (RoB 1.0). The research revealed 6 RCTs. The findings indicated that patients on anticoagulant therapy who received APCs without discontinuing their medication experienced a decreased post-operative bleeding, a shorter hemostasis time, reduced pain, and accelerated wound healing. However, due to the high/unclear risk of bias of the studies included, no definitive conclusions can be drawn on the superiority of APCs as hemostatic agents over other similar products. Additional studies are required to validate these findings.

## 1. Introduction

Hemostasis is the result of a set of well-regulated cellular and biochemical processes that maintain the blood in a liquid state in normal vessels and induce hemostatic clot formation after the occurrence of vascular damage. A congenital or iatrogenic (pharmacologically induced) deficiency in coagulation can be a serious risk in surgery.

Over the years, the population affected by cardiovascular diseases has been increasing. Antithrombotic drugs (antiplatelet and anticoagulant drugs) are commonly prescribed for the long-term prevention of arterial or venous thromboembolism (VTE) in cases of mechanical heart valves, atrial fibrillation, deep vein thrombosis, pulmonary embolism, coronary stents, and other clinical conditions with an increased thromboembolic risk [1].

In oral surgery, the treatment of patients on antithrombotic drugs is one of the most frequently encountered challenges. These patients are at a varying risk of thromboembolism and intra- and post-operative bleeding, depending on the pharmacodynamic characteristics of the anticoagulant drugs and on the type of surgical intervention [2].

If a patient treated with an oral anticoagulant must undergo invasive diagnostic or surgical procedures, the decision on how to manage the anticoagulant treatment requires a careful balancing act between the bleeding risk of the diagnostic/surgical procedure and the thrombotic risk resulting from any possible discontinuation of the anticoagulant.

There are many discrepancies relating to any interruption or modification of antithrombotic therapy in patients with cardiovascular disease. According to several clinicians, antiplatelet therapy should be stopped for patients with stable angina and stroke before dental extractions [3]. However, the current opinion recommends not interrupting therapy prior to surgery [4].

According to Appendix 3 of the Italian Drug Agency (AIFA) Note 97 and to the latest 2021 recommendations of the practical guide of the European Heart Rhythm Association (EHRA) [5,6], if a patient on AVK (anti-vitamin K drugs) is undergoing diagnostic/surgical procedures with a low or very low bleeding risk (such as the dental extraction of up to three teeth, periodontal surgery, and dental implant procedures where a good local hemostasis can be achieved), anticoagulant therapy can be continued. If a patient is undergoing diagnostic/surgical procedures with a high bleeding risk, it may be necessary to switch temporarily to low-molecular-weight heparins (“bridging”).

Today, thanks to the introduction of new oral anticoagulant drugs NOACs (non-vitamin-K oral anticoagulants) with their short half-life, more rapid onset and offset, fewer drug interactions, and the absence of any need for international normalized ratio (INR) assessment, this “bridging therapy” is no longer necessary [2]. The EHRA 2021 recommendations suggest that the patient characteristics (renal function, age, concomitant therapies and history of bleeding complications), the type of NOAC in use and the bleeding risk associated with the surgical procedure must be considered. Patients with a normal renal function can undergo surgical procedures with a low bleeding risk at least 24 h after taking the NOAC. Resumption of NOAC/direct oral anticoagulant (DOAC) therapy should not occur earlier than 24 h after the procedure unless otherwise indicated by the surgeon [7].

In patients undergoing low-risk hemorrhagic procedures where good local hemostasis is possible, the 2021 EHRA guidelines suggest that the NOAC should not be discontinued, taking advantage of the period of minimal drug action before assuming the next dose (in practice, the procedure can be scheduled 18–24 h after the last drug intake). Resumption of the NOAC is recommended 6–8 h after completion of the procedure [7].

Although many authors clearly indicate that the risk of bleeding is less even when multiple dental extractions must be performed at the same session [8,9,10] and that anticoagulated patients within therapeutic INR values (INR < 4.0) can safely undergo a tooth extraction without changing therapy [11,12,13], the use of hemostatic devices is recommended to stabilize or enhance clot formation at the surgical site [14,15,16]. In addition to digital compression, the surgeon should consider plugging with a sterile gauze soaked in saline solution (or tranexamic acid) and suturing, the most immediate local measure consisting of the use of hemostatic agents. Many studies have compared and evaluated different local hemostatic agents (oxidized cellulose [17], gelatin sponge [18], fibrin sponge [19], fibrin glue [20], cyanoacrylate glue [21], topical thrombin [22], epsilon-aminocaproic acid mouthwash [23], and tranexamic acid mouthwash [17,24]), showing that not all agents act in the same way or are equally effective in managing the risk of post-operative bleeding. However, several studies have affirmed that none of these hemostatic agents has shown superior results compared to the others [25,26,27].

During the past few years, the use of autologous platelet concentrates (APCs) in achieving an adequate post-operative hemostasis in patients with coagulation disorders has aroused great interest in the literature. APCs are blood products used in several medical and dental fields to increase the soft and hard tissue healing rate [28,29]. They represent a reservoir of growth factors which have been mostly involved in cell proliferation, chemotaxis, extracellular matrix production/angiogenesis [30,31,32], hemostasis, and the proliferative and remodeling phases of wound healing [33,34].

Several studies have shown that their use in post-extraction sockets without any modification of oral anticoagulant therapy leads to great results in the prevention of post-operative bleeding, i.e., a higher quality and more rapid post-operative tissue healing [32,35,36,37,38,39]. Nevertheless, there is no consensus regarding the superiority of APCs over other hemostatic agents.

Hence, the aim of this systematic review has been to evaluate the results of RCTs, comparing the hemostatic effect of APCs following dental extraction in patients assuming anticoagulant/antiaggregant medication with other hemostatic agents or when only suturing is performed. This review was compiled following the PRISMA (Preferred Reporting Items for Systematic Reviews and Meta-Analyses) guidelines [40] for improving the reporting of systematic reviews and meta-analyses.

## 2. Materials and Methods

In accordance with the PICO statement, this systematic review is aimed at answering the question “Does the use of APCs following dental extractions (Intervention/exposure) reduce the time to hemostasis (Outcome) in patients who are on anticoagulant/antiaggregant medication (Population)?” Other types of hemostatic agents and natural healing by blood clotting are considered as the Comparison/Control.

The primary outcome variable was the time to hemostasis. The secondary outcomes were the presence of post-operative bleeding, wound healing and biological complications (e.g., pain or infection).

The protocol was registered on the PROSPERO National Institute of Health Research Database (CRD42021258587).

### 2.1. Literature Search and Review Selection

Three electronic databases (PubMed, Scopus, and the Cochrane Central Register of Controlled Trials (CENTRAL)) were explored up to 31 March 2023 using a combination of keywords and MeSH terms according to the database rules (Table 1). A manual search was performed directly from the websites of the following scientific journals: the Journal of Clinical Periodontology, the Journal of Periodontology, the Journal of Periodontal Research, the International Journal of Periodontics and Restorative Dentistry, Clinical Oral Investigation, Clinical Oral Implant Research, International Surgery, Implant Dentistry, Quintessence International, the Journal of Prosthodontics, the International Journal of Prosthodontics, and the European Journal of Oral Implantology. An exploration of the grey literature was performed by searching among the conference abstracts published on the Web of Science and Scopus and on the databases of scientific dental congresses. Two authors (MDC, AA) carried out the screening of the articles, matching the search strategy using the Rayyan software. The eligibility criteria were: RCTs addressing the hemostatic effect of platelet-rich fibrin (PRF) treatment in patients undergoing anticoagulant/antiaggregant therapy; articles written in English; and articles published up to 31 March 2023. Controlled clinical trials (CCTs), cross-sectional studies, case series, questionnaires, radiographic studies, studies with histological data only, animal studies and case reports were excluded. Articles written in any language other than English were also excluded.

After title and abstract screening, the articles were selected for full-text reading. Whenever differences in the judgment of the eligibility of the title and abstract occurred, full texts were included for the final assessment.

In order to identify unpublished or discontinued studies, all authors of the selected studies were contacted and the bibliographies of all the selected studies and relevant reviews were checked.

Disagreements between the two investigators were solved through discussion; if needed, a third operator (GS) was contacted for a final decision.

### 2.2. Data Extraction

The data were independently extracted by two authors (RG and AA) using a pre-determined extraction form. Any disagreements were solved by discussion. All the authors were contacted to clarify information or identify missing information and, where no agreement was reached, the data were excluded until further clarification was possible. The following data were extracted: author, publication year, country of origin, total number of subjects included, participant data (age and gender), type of therapy, type of intervention, APC specifications, control groups, follow-up, outcomes evaluated, results and conclusions.

### 2.3. Risk of Bias Assessment

The risk of bias assessment was conducted using the risk of bias tool (RoB 1.0), the recommended approach for assessing the risk of bias in studies included in Cochrane reviews [Higgins JPT, Green S (eds) in accordance with the Cochrane Handbook for Systematic Review of Intervention 5.1.0 [updated March 2011] www.cochrane-handbook.org, accessed on 27 February 2019]. It is a two-part tool, addressing six specific domains (namely sequence generation, allocation concealment, blinding, incomplete outcome data, selective outcome reporting, and “other issues”). Each domain includes one specific entry in a “risk of bias” table. Within each entry, the first part of the tool involves describing what was reported to have happened in the study. The second part involves assigning a judgment relating to the risk of bias for that entry. This is achieved by answering a pre-specified question about the adequacy of the study in relation to the entry, such that a judgment of “Yes” indicates a low risk of bias, “No” indicates a high risk of bias and “Unclear” indicates an unclear or unknown risk of bias.

The methodological quality of the RCTs included was independently assessed by two reviewers (MDC and AA) as a stage of the data extraction process. In any case in which the study to be assessed had one or more review authors in the author list, it was independently evaluated only by those review authors not involved in the trials. After considering possible additional information provided by the authors of the trials, the studies were grouped into the following categories. It was assumed that the risk of bias was the same for all outcomes and each study was assessed as follows:

- (A) Low risk of bias (plausible bias unlikely to seriously alter the results) if all the criteria were met.
- (B) Unclear risk of bias (plausible bias that raises some doubt about the results) if one or more key domains had an unclear risk of bias.
- (C) High risk of bias (plausible bias that seriously weakens confidence in the results) if one or more criteria were not met.

### 2.4. Assessing the Certainty of the Evidence

Two review authors (MDC, AA) independently assessed the certainty of the evidence for the outcomes (time to hemostasis, post-operative bleeding, pain, and wound healing) according to the GRADE approach (Grading of Recommendations Assessment, Development and Evaluation Working Group) [41], which classifies the evidence based on five domains: methodological limitations (risk of bias), inconsistency, indirectness, imprecision, and publication bias. The quality of the evidence will be graded using “very low”, “low”, “moderate”, or “high”.

## 3. Results

### 3.1. Search Results

**Figure 1:**
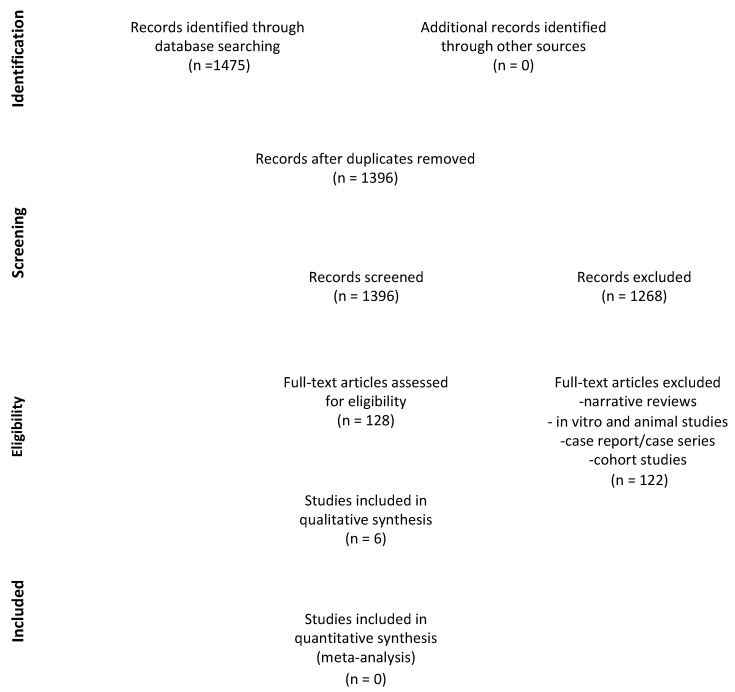
PRISMA Flow Diagram.

Figure 1 shows the flow diagram of the study selection. A total of 1475 studies were identified through electronic databases (PubMed, Scopus and the Cochrane Central Register of Controlled Trials (CENTRAL)). No studies were selected through other sources. After removing duplicates, the titles and abstracts of 1396 articles were analyzed. Subsequently, 128 articles were included for full-text eligibility, but 114 of these were excluded in accordance with the predetermined exclusion criteria. The most frequent exclusion criterion was the absence of an RCT model. Finally, six RCTs met the inclusion criteria and were included for the qualitative analysis [42,43,44,45,46,47]. A meta-analysis was not performed due to the heterogeneity in the data collected from the selected studies, such as variations in patient populations, treatment protocols, number of extractions and patient comorbidities, as well as the small number of studies included.

### 3.2. Characteristics of the Studies Included

The data extracted from the six RCTs are summarized in Table 2 and Table 3. Three studies [41,43,44] were conducted in Italy: two came from the same group in the Dental Clinic of the University “Magna Graecia” of Catanzaro [42,44], while one was conducted [45] in the San Sebastiano Hospital in Caserta. Two studies were conducted in India [46,47], in the University of Ambala, Haryana [46] and the University of Bangalore, Karnataka [46]. One study was realized in collaboration with the team of the Alexandria University in Egypt. Three studies had a parallel-group design [42,43,45,46,47], while only one study [43] had a split-mouth design. The number of total subjects included in each study was always specified and ranged from 20 to 300 patients differently divided into various groups of analysis. The patients’ ages were reported in all of the included RCTs: the means ranged from 46 to 64 years. Only in one study [47] was the number of males and females included not reported. Additionally, the type of anticoagulant therapy the patients had received was not always specified [44,46,47]. The categories of interventions included both single [43,45,46,47] and multiple dental extractions [42,44] relating to maxillary and mandibular, anterior, and posterior teeth. All of the included studies reported the APC preparation protocol and the specific use of the APCs. The APCs were compared to other hemostatic agents or to healing provided by blood clotting alone (only suturing). In most of the RCTs included the time to hemostasis and the presence of post-operative bleeding and pain had been assessed as primary outcomes. Other outcomes were the presence of wound healing and post-surgical complications.

### 3.3. Summary of Clinical Findings

#### 3.3.1. Time to Hemostasis

According to Sakar et al. [46], who analyzed the time to achieve hemostasis in patients treated with oral anti-platelet drugs due to prosthetic heart valves, rheumatic heart disease or previous myocardial infarction, with INR values between 1 and 3.5, PRF gel presented a shorter time to achieve hemostasis (mean of 1.18 min) compared to chitosan gel (mean of 2.64 min). According to Giuffrè et al. [45], in patients undergoing therapy based on dicumarol medication, the use of PRP in the post-extraction socket showed an excellent hemostatic capacity with results comparable to the control group (patients who stopped the therapy and replaced it with calciparin). Thus, the duration of the hospital stay was reduced, and the risk of thromboembolism was eliminated. Similar findings were observed by Eldibany [43], who reported no statistically significant difference in the time to achieve hemostasis between the use of PRF and HemCon dental dressing (47.6 s vs. 51.3 s).

#### 3.3.2. Post-Operative Bleeding

Brancaccio et al. [42], using the classification proposed by Souto [23], evaluated the post-operative bleeding assessed 30 min after the extractions and showed that L-PRF and A-PRF+ were linked to a significant reduction in the bleeding risk compared to the control group (suturing) and hemostatic plug (HEM) groups. Brancaccio et al. [42] have also been recording information about vicious habits (cigarette smoking and alcohol consumption) and pathological anamnesis. Patients with hypertension and diabetes showed an increased risk of bleeding.

#### 3.3.3. Pain Score and Wound Healing

Sarkar and Brancaccio [42,46] also evaluated the pain score and wound healing after dental extractions. Thanks to the presence of leukocytes and certain growth factors realized during the centrifugation, PRF could be assessed as a great method to reduce post-operative infection and pain. Thus, a good post-extraction site healing on day 7 can be guaranteed (80% of sites in group A and 60% of sites in group B), demonstrating the superior efficacy of PRF gel. At the same time, L-PRF and A-PRF+ showed better healing than the control and HEM groups. Nevertheless, smoking and diabetic patients were associated with a higher probability of delayed healing with an increased risk of post-extraction alveolitis [42]. However, Giudice et al. [44] did not report any statistically significant difference in terms of wound healing evaluated with the Friedman test. No post-operative bleeding and minimum pain occurred on the day of surgery and on the following days (VAS average 2) and no cases of alveolitis were reported in the PRF group compared to the group treated by suturing alone. Only Rajendra et al. [47] demonstrated that the chitosan based axiostat hemostatic agent seems to be a superior wound dressing material in achieving hemostasis and managing post-operative pain in patients on anticoagulant therapy. Nevertheless, those findings were not so statistically significant compared to the PRF group.

### 3.4. Risk of Bias of the Included Studies

**Figure 2:**
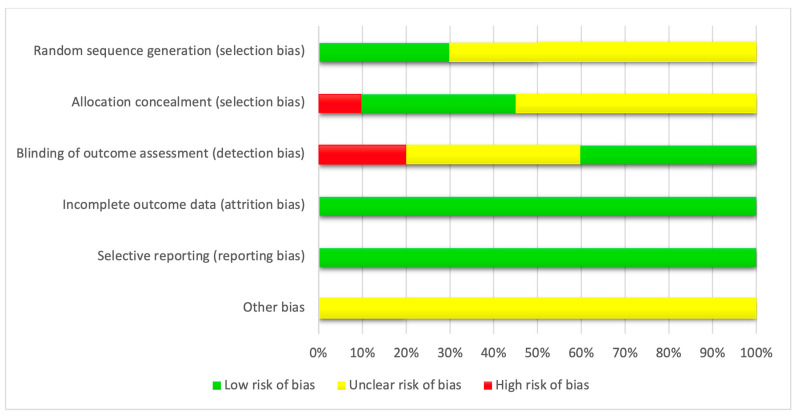
Risk of bias graph: review authors’ judgments about each domain, expressed in percentages, of all included studies.

**Figure 3:**
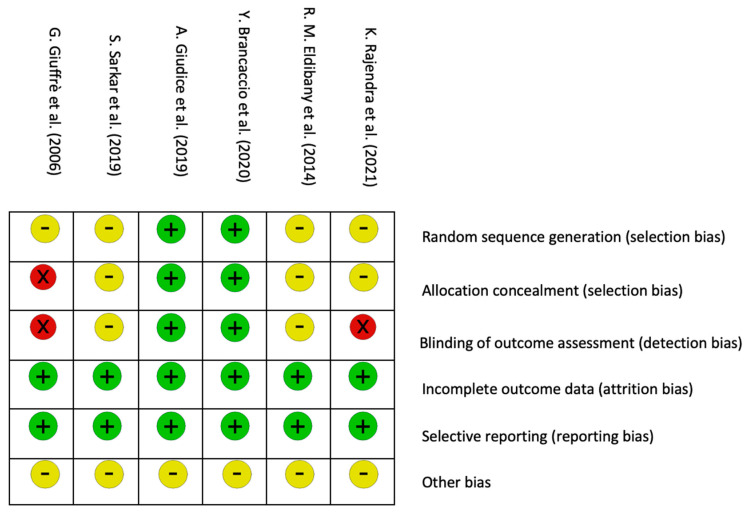
Risk of bias summary: judgments of the review authors on each domain of all included studies [42,43,44,45,46,47].

The final risk of bias assessment is summarized in Figure 2 and Figure 3 and Table S1. It was not necessary to ask for any unclear or missing information from the trial authors since all the information was reported in the publications. Each trial was assessed as having a low, unclear or high risk of bias. Two trials were assessed as high risk [45,47], while the others were assessed as unclear risks [42,43,44,46].

### 3.5. Certainty of the Evidence

The certainty of evidence for the main comparison (APCs versus other hemostatic agents or physiological healing) was classified as low for time to hemostasis which represents that APCs may make little or no difference compared to control. On the contrary, the certainty of evidence for the main comparison was classified as moderate for post-operative bleeding, pain, and wound healing, which indicates that the likelihood that the effect of APCs will be substantially different compared to the control is moderate. There was a downgrade in the levels of evidence due to the methodological quality of the studies, the different types of APCs analyzed, and the small sample size. Table 4 shows the summary of findings of GRADE assessment.

## 4. Discussion

The aim of this paper has been to review systematically the evidence concerning the hemostatic effectiveness of APCs following dental extraction in patients taking anticoagulant/antiaggregant medication without discontinuing the therapy.

The findings indicate that these patients experienced a decreased post-operative bleeding, shorter time to achieve hemostasis, reduced pain and accelerated wound healing.

Cross-linked fibrin clot formation is essential to avoid hemorrhages. The tridimensional fibrin network allows for a clotting stability in the last phase of the coagulation process by ensuring the regeneration of injured tissues [48,49]. In fact, thanks to the fibrin structure, blood clotting presents mechanical properties to contrast arterial pressure and safeguard the integrity of the damaged vessel and the whole healing process [50]. Antithrombotic drugs, restricting both platelet aggregation (antiplatelets) and the activation of clotting factors (dicoumarols), results in a delay of fibrin clot formation [51,52].

Local hemostats are sterile medical devices, which can be derived from plants (polysaccharides and cellulose derivatives), animals (collagen and gelatins), or minerals (zeolite: only surgically removable). The mechanism of action is chemical and/or mechanical. Hemostats promote platelet aggregation on the surface, creating a substrate for the coagulation cascade. To date, several hemostatic agents used in oral and periodontal surgery have been studied in the literature, in particular, oxidized regenerated cellulose [53]; resorbable gelatin [54]; collagen [55]; lysine analogues, serine protease inhibitors, and fibrin sealants [18]; fibrin glue [56]; cyanoacrylate-based glues [57]; human thrombin and thrombin gelatin-matrix [58]; tranexamic acid [59,60]; and chitosan [61]. Although these local hemostats have shown numerous advantages over the years, such as ease of use (flexibility and malleability), adaptability to a wide range of surgical procedures, rapid and complete resorption (within 7–14 days), and bacteriostatic action, they also have several disadvantages, including high cost, inflammatory reactions, and the lack of any autologous origin, which could lead to localized immunogenic reactions.

Nowadays, the development of APC technologies offers simplified and optimized products of an autologous origin without any production costs. APCs form a natural fibrin matrix, which acts as a scaffold at the wound site. The fibrin matrix helps to stabilize the clotting, promote platelet adhesion, and provide a physical barrier to prevent further bleeding. Moreover, APCs contain a large quantity of platelet growth factors, such as platelet-derived growth factor (PDGF), transforming growth factor-beta (TGF-β), and vascular endothelial growth factor (VEGF), and cytokines, which stimulate the healing process, promote tissue regeneration, enhance the formation of new blood vessels and reduce post-operative pain [34,37,62].

APCs exhibit variations in the polymerization and final fibrin architecture, which may influence the strength and the growth factor trapping/release potential of the clotting with diverse effects on the process of hemostasis. For example, during the PRF preparation, the polymerization of fibrinogen into fibrin occurs slowly, naturally and progressively in the presence of physiological thrombin, resulting in a strong natural fibrin meshwork, composed of a tetra molecular structure. On the contrary, for PRP products, bilateral junctions of the fibrin fibrillae are provoked by a drastic activation and polymerization, for example, with high thrombin concentrations, which leads to a dense network of monofibers, similar to a fibrin glue [31].

The recent literature has indicated that there is no significant difference in post-extraction bleeding among patients on an uninterrupted therapy with various antiplatelet/anticoagulant medications. DOACs, as well as single or dual-antiplatelet medications, showed the same incidence of post-operative bleeding and determined an acceptable rate of controllable post-operative bleeding after a simple tooth extraction [62]. Therefore, they can safely be used without interrupting the therapy and adopting local hemostatic measures [11,26,63,64,65].

In fact, the RCTs included in this review showed that continuing the therapy was linked to a post-operative bleeding which did not cause any serious adverse complications and was easily managed using hemostatic agents and instructions given to the patients.

While the included studies are valuable randomized, controlled trials (RCTs), which are widely regarded as the gold standard for clinical trials, it is important to note that a meta-analysis, which combines numerical results from multiple similar studies, was not conducted. This omission was due to the heterogeneity observed in the data collected from the selected studies, such as variations in the patient populations, treatment protocols, number of extractions, and patient comorbidities. In fact, the included RCTs considered different types of control groups using various hemostatic agents, such as hemostatic sponges and tranexamic acid [45] or chitosan [43,46,47]. In addition, there were no restrictions regarding the inclusion of all types of APCs and the related methods of preparation, which can influence the macroscopic characteristics of the APCs and their biological properties (different percentages of platelets, leukocytes, growth factors, and the fibrin matrix) and, consequently, can have an impact on the final outcomes.

Another factor which affected the heterogeneity of the studies and the assessment of both the primary and secondary outcomes is the number of teeth extracted. In Brancaccio et al. [42] and Giudice et al. [44], multiple tooth extractions (at least four non-adjacent tooth elements) were planned for each patient, while in Sarkar et al. [46], Rajendra et al. [47], Giuffrè et al. [45], and Eldibany et al. [43], single extractions (of anterior or posterior tooth elements) were performed. Additionally, although the position of the tooth to be extracted may be a further element to consider, the included studies did not specify this. This is another factor, therefore, which could influence the complexity of the surgery and so the result in terms of post-operative conditions.

Furthermore, it must be taken into account that patient factors, such as the presence of comorbidities and vicious habits, may influence post-operative bleeding and wound healing [66]. Only Brancaccio et al. considered vicious habits (cigarette smoking) and comorbidities (hypertension and diabetes) which can increase the likelihood of bleeding and delayed wound healing beyond 30 min post-operatively. From a comparison of the data, the results show that patients with diabetes and/or hypertension had a greater risk of bleeding while, diabetes and smoking were associated with a slower healing process. In diabetic patients, a poor wound healing is not uncommon after surgery due to a decrease in collagen and a reduced secretion of growth factors. The hyperglycemic status increases the risk of developing complications due to the altered cell response and the set of activated inflammatory cytokines. Smoking may also interfere with soft tissue healing after oral surgery with a dose-dependent effect. The mechanism is not yet well known; however, the presence of nicotine and carbon monoxide may be one of the causes. Nicotine has been shown to increase the risk of vasoconstriction and decrease tissue perfusion followed by vascular occlusion [21,67]. Thus, regardless of the choice of the hemostatic agent, it is necessary to evaluate comorbidities and vicious habits, which could play a role in post-operative bleeding and wound healing.

Furthermore, it is critical to highlight that the most recent oral anticoagulants (such as apixaban, edoxaban, and rivaroxaban) were not considered in the selected studies. This deficiency makes it problematic to assess the effective role of APCs in patients assuming NOACs, even if their effectiveness could be hypothesized through a better management of these drugs compared to dicoumarol.

In conclusion, we advise caution when interpreting the findings of the present systematic review, taking into consideration the above-mentioned limitations.

## 5. Conclusions

The findings indicate that patients on anticoagulant therapy who received APCs without discontinuing their medication experienced decreased post-operative bleeding, shorter time to achieve hemostasis, reduced pain, and accelerated wound healing. However, due to the high/unclear risk of bias of the included studies, no definitive conclusions can be drawn on the superiority of APCs as hemostatic agents over other forms of medication and no valid clinical guidelines for the use of APCs as a hemostatic agent can be proposed. For this reason, an additional aim of this paper is to raise awareness in the scientific community to encourage researchers to explore the role of the APCs, also in patients taking NOACs, and to perform further studies which could provide more homogeneous results qualitatively and quantitatively which might be comparable and subject to meta-analysis.

## Figures and Tables

**Table 1:** Search strategy.

Pubmed	(“tooth extraction”[MeSH Terms] OR “dental extraction*”[All Fields] OR “dental avulsion”[All Fields] OR “hemorrhagic risk” [Title]) AND (“platelet-rich plasma”[Title/Abstract] OR “platelet-rich fibrin”[Title/Abstract] OR “PRF”[Title/Abstract] OR “PRP”[Title/Abstract] OR “antiplatelet therapy”[Title/Abstract] OR “anticoagulant therapy”[Title/Abstract] OR “antiplatelet drugs”[Title/Abstract] OR “platelet rich in growth factors”[Title/Abstract] OR “anticoagulant drugs”[Title/Abstract] OR “autologous platelet concentrates”[Title/Abstract] OR “hemocomponents”[Title/Abstract] OR “concentrated growth factors”[Title/Abstract] OR “CGF”[Title/Abstract] OR “hemostasis”[Title/Abstract])
Scopus	(TITLE-ABS-KEY (tooth extraction*) OR TITLE-ABS-KEY (dental extraction*)) AND (TITLE-ABS-KEY (“ platelet-rich plasma “) OR TITLE-ABS-KEY (“platelet-rich fibrin”) OR TITLE-ABS-KEY (“PRP”) OR TITLE-ABS-KEY (“PRF”) OR TITLE-ABS-KEY (“antiplatelet therapy”) OR TITLE-ABS (“anticoagulant therapy”) OR TITLE-ABS-KEY (“ autologous platelet concentrates “) OR TITLE-ABS-KEY (“anticoagulant drugs”) OR TITLE-ABS-KEY (“antiplatelet drugs”) OR TITLE-ABS-KEY (“ concentrated growth factors”) OR TITLE-ABS-KEY (“ CGF “) OR TITLE-ABS-KEY (“ hemocomponents “) OR TITLE-ABS-KEY (hemostasis))
CENTRAL	(hemostasis in tooth extraction)

**Table 2:** Study characteristics: HEM, hemostatic plug; L-PRF, leucocyte–platelet-rich fibrin; A-PRF, advanced platelet-rich fibrin; HDD, HemCon dental dressing; VAS, visual analogue scale; PRP, platelet-rich plasma.

Author, Year of Publication	Country	Number of Patients	Age (Mean) Range)	Gender (M, F)	Patients’ Characteristics	Intervention	APC Specifications	Control	Follow-Up	Outcomes
**Brancaccio, 2020 [42]**	Magna Graecia University of Catanzaro, Italy	102 patients divided into 4 groups: Control group (suturing alone) and 3 different hemostatic agents—HEM group, L-PRF group and A-PRF group (test groups).	64.1 ± 17.4	74; 28	Patients undergoing long-term treatment with oral antiplatelets: acetylsalicylic acid 100 mg (72 patients), Clopidogrel 75 mg (15 patients), acetylsalicylic acid 100 mg + clopidogrel 75 mg (6 patients), Ticagrelor 90 mg (5 patients) and ticagrelor 90 mg + acetylsalicylic acid 100 mg (4 patients).	All patients were subjected to 4 dental extractions of non-adjacent teeth at the same time and to the application of different hemostatic agents (A-PRF+, L-PRF, HEM).	For A-PRF+, two test tubes were used, corresponding to 18 mL of venous blood, which were centrifuged at 1300 rpm for 8 min in the DUO PRF centrifuge. For L-PRF, two test tubes were used, corresponding to 12 mL of blood, which were centrifuged at 2700 rpm for 18 min using the IntraSpin centrifuge.	Natural healing using suturing alone	30 min (frequency of post-operative bleeding), 2 weeks (healing of the surgical wound after the extraction)	Post-operative bleeding (Souto classification), any possible complications and wound healing
**Eldibany R.M. et al., 2014 [43]**	Alexandria University, Egipt	20 patients divided into 2 groups: group A (test group = 10 patients) where PRF was inserted into the extraction socket and group B (control group: 10 patients) where the extraction socket was packed by HDD.	46.65; 36–62	11; 9	All patients were cardiac patients and had undergone heart valve replacement and were currently taking warfarin.	All patients required extraction of a single mandibular posterior tooth. The extractions were performed for all patients without altering the dose of the anticoagulant and in the test group the extraction sockets were packed with PRF.	For the PRF preparation, 20 mL of blood were collected without any anticoagulant from the brachial vein 12 min before the extraction. The blood was transferred and equally divided into two 10 mL sterile glass tubes and was immediately centrifuged using a table centrifuge at 3000 rpm for 12 min.	HemCon dental dressing (HDD) 10 mm × 12 mm, a hemostatic agent.	1–7 days	Duration of bleeding and pain (VAS)
**Giudice A. et al., 2019 [44]**	Magna Graecia University of Catanzaro, Italy	40 patients equally divided into 4 groups: suturing alone (control group = 10 patients) and suturing + hemostatic plug HEM (10 patients) suturing + A-PRF+ (10 patients), suturing + L-PRF into the socket (10 patients) as test groups.	60.9; 18–88	28;12	Patients undergoing oral antiplatelet therapy (type of drug not specified).	For all patients requiring the extraction of at least four non-adjacent teeth (incisors, canines, premolars, and molars). After the extractions, hemostatic plugs (HEM) were put in the sockets in the. A-PRF+ and L-PRF (test groups) without reducing the dose of oral antiplatelets.	For the A-PRF+, two tubes, corresponding to 18 mL of blood, were sampled and centrifuged immediately at 1300 rpm for 8 min using the PRF DUO centrifuge. For the L-PRF, two tubes corresponding to 12 mL of blood were sampled and centrifuged immediately for 18 min at 2700 rpm using the IntraSpin centrifuge.	Natural healing using suturing alone	30 min for the frequency of post-operative bleeding and 1 and 2 weeks post-extraction for the wound healing	Complications, time to complete each procedure, post-operative bleeding, costs of the materials, patient preference and wound healing index recorded 1 and 2 weeks post-extraction by blinded assessors
**Giuffrè G. et al., 2006 [45]**	St. Sebastian Hospital, Caserta, Italy	208 patients divided into 4 groups consisting of 52 patients each (A,B,C,D).	64.5; 51–68	84; 124	All patients were undergoing anticoagulant therapy (warfarin). The patients belonging to the first 3 groups (A, B, C), had undergone extractions without discontinuing the therapy. In group A (test group) PRP + suturing were used; in group B PRP, hemostatic sponges and suturing were used; in group C hemostatic sponges, suturing and compression by means of gauzes soaked in tranexamic acid were used; group D consisted of patients where dicumarol therapy had been suspended and replaced by heparincalcium.	All patients underwent oral surgical operations: simple tooth extractions, root extraction, and cystectomies. In group A (test group), the socket was filled with PRP only and sutured.	For the PRP preparation the blood (70 mL ± 30) was centrifuged at 180° for 10 min. so that plasma, which is full of platelets, was separated from the thick red corpuscle layer. The PRP obtained was then further centrifuged at 1800° for 10 min in order to obtain hyperconcentrated platelets.	1: PRP + hemostatic sponges; 2: hemostatic sponges and local tranexamic acid; 3: therapy modification	3, 12, 24, 48 h	Post-operative bleeding
**Rajendra K. et al., 2021 [47]**	University of Ambala, Haryana, India.	300 patients divided into two categories (n = 150, respectively) as Group I (PRF dressing—test group) and Group II (Axiostat dressing).	52.5; 35–70	not specified	All patients had a medical history of cardiac disease and were on anti-platelet drug therapy (type of drug not specified).	Extraction of single tooth filled with PRF (test group).	PRF membrane was placed after preparing it in clean and sterile glass test tubes using centrifugation at 3000 rpm for 10 min.	Axiostat (chitosan)	7 days post-operative	Duration of bleeding and pain
**Sarkar S, 2019 [46]**	University of Bangalore, Karnataka, India	60 patients allocated equally in two groups: group A (test group), where PRF gel was packed into the extraction socket, while in group B (control group), chitosan hydrogel was packed.	58.77; 35–82	29; 31	Patients undergoing oral antiplatelet therapy (type of drug not specified).	For all patients requiring the extraction of a single tooth, after the extractions PRF was put in the sockets (test group).	PRF gel was prepared in patients of Group A f(test group) with 5 mL of blood that was drawn from the brachial vein of each patient and was centrifuged at 3000 rpm for 10 min.	Chitosan	1, 3, 7 days	Time to hemostasis, pain score, alveolar osteitis, secondary hemorrhage and healing

**Table 3:** Study characteristics: HEM, hemostatic plug; L-PRF, leucocyte–platelet-rich fibrin; A-PRF, advanced platelet-rich fibrin; HDD, HemCon dental dressing; VAS, visual analogue scale; PRP, platelet-rich plasma.

Author, Year of Publication	Results	Conclusion
**Brancaccio, 2020 [42]**	Bleeding sites were in numbers of 20, 12, 2, and 5 for the suture-only group, for the HEM group, for the A-PRF+ group, and for the L-PRF group, respectively. Instead, the sites not completely healed 2 weeks after the extraction were in numbers of 31, 39, 22, and 15 for the suture-only group, for the HEM group, for the A-PRF+ group, and for the L-PRF group, respectively.	L-PRF and A-PRF represent a valid alternative to the traditional hemostatic treatment, reducing post-surgical bleeding and promoting wound healing.
**Eldibany R.M. et al., 2014 [43]**	Regarding the time to hemostasis in the test group the mean time was 47.6 ± 1.3 s while in the control group the mean time was 51.3 ± 2.1 s. Regarding the pain in the test group there was no notable post-surgical pain (VAS average 2, ranging between 1 and 3) on the 2nd post-extraction day and 0 on the following days. Instead, in the control group, four cases reported severe pain (VAS = 8) in the first 48 h following the extraction, while two cases showed post-operative pain of VAS 5 on the first two post-extraction days.	PRF has good anti-hemorrhagic properties and increases tissue healing and wound closure, thus allowing for a quick recovery without significant painful events.
**Giudice A. et al., 2019 [44]**	Two weeks after the extraction, no patient dropped out and no complication was reported. The average time to complete suturing after tooth extractions was: 1.0 ± 0.00 min at the control sites, 1.5 ± 0.41 at the HEM sites, 2.8 ± 0.61 at the A-PRF+ sites, and 2.8 ± 0.56 at the L-PRF sites. Post-operative bleeding 30 min after the extraction was present at 8, 5, 1 and 2 sites for the control, HEM, A-PRF+ and L-PRF sites, respectively. One week after the extraction the mean wound healing index was 1.05 ± 0.60 for the control, 1.18 ± 0.59 for the HEM, 1.00 ± 0.68, A-for the PRF+ and 0.95 ± 0.50 for the L-PRF sites. Two weeks after the extraction the mean wound healing index was 0.33 ± 0.53 for the control, 0.43 ± 0.50 for the HEM, 0.25 ± 0.49 for the A-PRF+ and 0.15 ± 0.36 for the L-PRF sites. One week after the extraction, nine patients preferred control sites, eight HEM, ten A-PRF+ four L-PRF and nine had no preference. Costs without counting sutures and blood centrifuges were 0.00, 14.49, 2.44 and 2.44 euros for the control, HEM, A-PRF+ and L-PRF sites, respectively.	A-PRF+ was associated with less post-operative bleeding when compared to suturing alone.
**Giuffrè G. et al., 2006 [45]**	The number of hemorrhages in the test group (group A) was 0 at 3 and 12 h, 2 at 24 h and 0 at 48 h. Patients belonging to the groups A and B showed a very good hemostasis compared to the patients of group D. As for group C (19 males), 6 patients (i.e., 11.5%) showed a good hemostasis, both immediately and in the long term, so that they could be discharged on day 2 after surgery.	The results strongly encourage using PRP regularly when carrying out surgical treatments on patients who are undergoing a coumarin therapy.
**Rajendra K. et al., 2021 [47]**	The average pain score was 1.86 ± 0.06 in Group I (test group) and 1.05 ± 0.87 in Group II (control group). Thus, a lower post-operative pain was seen with Axiostat dressing. Hemostasis was achieved in Group II participants in 1.25 ± 0.06 min and in Group I patients 1.89 ± 0.54 min.	Chitosan is a superior wound dressing material in achieving hemostasis in cardiac patients on antiplatelet medication after tooth extraction.
**Sarkar S, 2019 [46]**	All the extraction sockets with Platelet-rich fibrin achieved hemostasis in 2.64 min and sockets with Chitosan hydrogel achieved hemostasis in 1.182 min (*p* < 0.001). Post-operative pain in Group A sites (3.2, 1.4, 0.37 on 1st, 3rd & 7th day respectively) was significantly lower than the control sites (3.4, 1.67, 0.53 on 1st, 3rd & 7th day respectively). A total of 4 patients, 2 in each group, reported alveolar osteitis on the 7th day after extraction. There was a statistically significant (*p* < 0.001) better healing at the Group A extraction sites. 80% of the patients of Group A showed a healthy healing (score of 5) of the extraction sockets based on the custom-made evaluation chart by 7th post-operative day.	Chitosan hydrogel dressing thus proved to be a superior hemostatic agent compared to PRF gel, significantly shortening the clotting time following dental extraction in patients under antiplatelet therapy. However, PRF gel has superior wound healing properties compared to Chitosan with less postoperative pain following minor oral surgical procedures under local anesthesia.

**Table 4:** Summary of findings of GRADE assessment.

**Patients or population: ^1^** Patients undergoing anticoagulant therapy. **Settings: ^1^** Dental Clinic of the University “Magna Graecia” of Catanzaro, San Sebastiano Hospital in Caserta; University of Ambala, Haryana; University of Bangalore, Karnataka; Alexandria University in Egypt. **Intervention: ^1^** Use of autologous platelet concentrates (APCs) to gain hemostasis. **Comparison: ^1^** Other hemostatic agents or physiological healing.			
**Outcomes ^2^**	**Impact ^3^**	**Number of participants(Studies) ^4^**	Certainty of the evidence (GRADE) * ^5^
**Time to hemostasis**	APCs had a shorter time to achieve hemostasis (mean 1.18 min) than the control group (mean 2.64 min).	60 (1 study)	⊕⊕⊖⊖ Low
**Post-operative bleeding**	APCs provided for a significantly reduction of the bleeding risk compared to the control group.	102 (1 study)	⊕⊕⊕⊖ Moderate
**Pain score**	APCs seem to be a great method to reduce post-operative infection and pain.	442 (3 studies)	⊕⊕⊕⊖ Moderate
**Wound healing**	APCs provided for a better and faster wound healing.	142 (2 studies)	⊕⊕⊕⊖ Moderate
* GRADE Working Group grades of evidence: **High** = This research provides a very good indication of the likely effect. The likelihood that the effect will be substantially different ^†^ is low. **Moderate** = This research provides a good indication of the likely effect. The likelihood that the effect will be substantially different ^†^ is moderate. **Low** = This research provides some indication of the likely effect. However, the likelihood that it will be substantially different ^†^ is high. **Very low** = This research does not provide a reliable indication of the likely effect. The likelihood that the effect will be substantially different ^†^ is very high. ^†^ Substantially different = a large enough difference that it might affect a decision.			

^1^ The characteristics of the evidence, including the types of participants (patients or populations), types of settings (e.g., countries) where the studies were performed, the intervention, and what the intervention was compared to. ^2^ The most important outcomes, including the intended benefits, possible harms and costs. ^3^ The estimated impact of the intervention on each outcome (preferably provided quantitatively). ^4^ The amount of information upon which the information is based, such as the number of participants or units (e.g., facilities), as well as the number of studies. ^5^ The quality of the evidence for each outcome.

## Data Availability

No new data were created or analyzed in this study. Data sharing is not applicable to this article.

## Supplementary Materials

The following supporting information can be downloaded at: https://www.mdpi.com/article/10.3390/jcm12165342/s1, Table S1: Risk of bias summary: judgments of the review authors on each domain of all the included studies.
