# Supplementary material for: The Effectiveness and Safety of Autologous Platelet Concentrates as Hemostatic Agents after Tooth Extraction in Patients on Anticoagulant Therapy: A Systematic Review of Randomized, Controlled Trials
Source: J Clin Med. 2023 Aug 17;12(16):5342. doi: 10.3390/jcm12165342 (PMC10455824; doi:10.3390/jcm12165342)
Supplement: Supplementary file 1 [file jcm-12-05342-s001.zip › jcm-2499996-supplementary.pdf]

Table S1. Risk of bias summary: judgments of the review authors on each domain of all the included studies.

**Giuffrè et al. 2006**	**Authors' judgement**	**Support for judgement**
Random sequence generation	Unclear risk	"The above patients were divided at random into 4 groups…"
Allocation concealment	High risk	Not reported
Blinding of outcome assessment	High risk	Not reported
Incomplete outcome data	Low risk	Not reported
Selective reporting	Low risk	All outcomes specified in Methods have been reported.
Other bias	Low risk	The study appears to be free of other source of bias
**Sarkar et al. 2019**	**Authors' judgement**	**Support for judgement**
Random sequence generation	Unclear risk	"Subjects were randomly allocated into two groups…"
Allocation concealment	Unclear risk	"Patients were allocated equally in two groups; Group A: where PRF gel was packed into the extraction socket, while Group B: Chitosan hydrogel was packed."
Blinding of outcome assessment	Unclear risk	Not described in sufficient detail
Incomplete outcome data	Low risk	Not reported
Selective reporting	Low risk	All outcomes specified in Methods have been reported.
Other bias	Low risk	The study appears to be free of other source of bias
**Brancaccio et al. 2020**	**Authors' judgement**	**Support for judgement**
Random sequence generation	Low risk	"One computer-generated restricted random list was created with four treatments in random order…"
Allocation concealment	Low risk	"The random codes were enclosed in sequentially numbered, identical, opaque, sealed envelopes."
Blinding of outcome assessment	Low risk	"Only one of the investigators, not involved in the selection and treatment of the patients was aware... A dental practitioner with expertise in dental biostatistic analyzed data without knowing group allocation… These evaluations were performed by a second blinded outcome assessor…"
Incomplete outcome data	Low risk	"All enrolled patients concluded the study, and no drop-out occurred"
Selective reporting	Low risk	All outcomes specified in Methods have been reported.
Other bias	Low risk	The study appears to be free of other source of bias.
**Giudice et al. 2019**	**Authors' judgement**	**Support for judgement**
Random sequence generation	Low risk	"One computer-generated restricted random list was created. The list had four treatments in random order…"
Allocation concealment	Low risk	"Identical, sequentially numbered envelopes containing the random codes were used."
Blinding of outcome assessment	Low risk	"One independent investigator not involved in patient's treatment and selection had access to the randomized list… One investigator was responsible

		for generating the random allocation process…All outcome measures were assessed by a single blinded outcome assessor…”
Incomplete outcome data	Low risk	“No patient dropped out and data of all patients were evaluated in the statistical analysis”
Selective reporting	Low risk	All outcomes specified in Methods have been reported.
Other bias	Low risk	The study appears to be free of other source of bias.
**Eldibany et al. 2014**	**Authors’ judgement**	**Support for judgement**
Random sequence generation	Unclear risk	“The patients were randomly divided into two groups….”
Allocation concealment	Unclear risk	“Patients were allocated equally in two groups; group A: where PRF ..”
Blinding of outcome assessment	Unclear risk	“The allocation of patients into either group was random non blind as there was… ”
Incomplete outcome data	Low risk	Not reported
Selective reporting	Low risk	All outcomes specified in Methods have been reported.
Other bias	Low risk	The study appears to be free of other source of bias
**Rajendra et al. 2021**	**Authors’ judgement**	**Support for judgement**
Random sequence generation	Unclear risk	“The study participants were randomly divided into two groups ..”
Allocation concealment	Unclear risk	“Two groups containing equal numbers of participants, i.e., n = 150, each in Group I (PRF) and Group II (Axiostat), respectively.”
Blinding of outcome assessment	High risk	Not reported
Incomplete outcome data	Low risk	Not reported
Selective reporting	Low risk	All outcomes specified in Methods have been reported.
Other bias	Low risk	The study appears to be free of other source of bias
